# Examining the relationships among child care providers’ knowledge of child development, burnout, organizational climate, and expulsion risk: A brief report

**DOI:** 10.1002/imhj.70087

**Published:** 2026-04-26

**Authors:** Juan Wang, Cheri J. Shapiro, Tristan Collier, Jessica Sharp

**Affiliations:** ^1^ Institute for Families in Society, College of Social Work University of South Carolina Columbia South Carolina USA; ^2^ Institute for Families in Society, College of Social Work, Department of Psychology University of South Carolina Columbia South Carolina USA; ^3^ South Carolina Program for Infant/Toddler Care, College of Education University of South Carolina Columbia South Carolina USA

**Keywords:** burnout, early childhood expulsion, ECE workforce, knowledge of child development, organizational climate

## Abstract

Expulsion poses a significant barrier to high‐quality and inclusive early care and education (ECE), leading to both immediate and long‐term negative consequences for children and their families. This descriptive, exploratory study examined whether ECE providers’ knowledge of child development, burnout, and organizational climate was associated with their attitudes and perceptions related to expulsion risk. A total of 309 ECE providers in one state in the Southeastern region of the United States taking part in a program evaluation completed an online survey prior to program implementation. Multiple linear regression analyses were conducted to assess associations among providers’ knowledge of child development, burnout, organizational climate, and attitudes related to expulsion risk. Provider burnout was found to be significantly associated with providers’ attitudes related to expulsion risk (*β* = .36, *p* < .001) despite relatively modest levels of burnout reported by this sample. Organizational climate was significantly associated only with the hopelessness subscale of the expulsion risk measure used (*β* = –.21, *p* = .034). Findings are somewhat consistent with prior research and have implications for the field regarding methods to address ECE provider burnout and organizational climate to mitigate expulsion risk and promote more inclusive ECE environments.

## 1 INTRODUCTION

Early childhood expulsion is the permanent removal of a child from an early care and education (ECE) program. In the contemporary landscape of advancing access to and engagement with high‐quality ECE programs, the practices of expulsion emerge as significant impediments. The 2016 National Survey of Children's Health (NSCH) indicates that approximately 174,309 preschoolers were suspended, and 17,248 were expelled during the year 2016 alone (Zeng et al., 2019). Notably, preschoolers enrolled in state‐funded prekindergarten programs face expulsion rates (6.67 per 1000 enrollees) more than triple those of their older counterparts in grades K‐12 (2.09 per 1000 enrollees). Expulsion rates in child care and preschool settings are even higher, reaching 27.42 per 1000 students (Gilliam, 2005; Gilliam & Shahar, 2006).

The immediate and long‐term consequences of early childhood expulsion can be significant. When a child is expelled, they are excluded from environments designed to teach school readiness, depriving them of the benefits of early education. In the short term, children and their families experienced intense emotional distress, community isolation, financial strain, and difficulty finding alternative care, especially for children with disabilities (O'Grady et al., 2024). Although research on the long‐term effects of expulsion from ECE settings is limited, studies in K‐12 settings indicate that early expulsion is a predictor of future exclusionary discipline (Bowman‐Perrott et al., 2013). Over time, expelled students are significantly more likely to face negative academic outcomes, involvement with the justice system, and subsequent substance use (Anderson et al., 2019; Gerlinger et al., 2021; Prins et al., 2023).

Given the high prevalence and serious consequences of early childhood expulsion, it is crucial to identify factors that can help reduce its occurrence. A systematic review by Zinsser et al. (2022) found that exclusionary practices are influenced by multiple ecological systems, including microsystem (child and family factors), mesosystem (e.g., parent‐teacher interaction), and ecosystem (e.g., program types and resources and characteristics of administrators). Similarly, Miles et al. (2021) identified child, program, and community‐level factors simultaneously associated with expulsion practices among licensed child care providers. They found that at the child level, children's gender, age, and disability status have significant relationships with expulsion practices; at the program and community level, district affiliation, the use of Infant and Early Childhood Mental Health consultants, and access to quality coaches and community poverty ratios were associated with expulsion (Miles et al., 2021).

**Key Findings**

1. Higher levels of child care provider burnout were significantly associated with an increased risk of attitudes favorable to use of expulsion in ECE settings.
2. Child care providers’ perceptions of organizational climate were negatively associated with providers’ hopelessness about being able to improve children's behaviors.
3. Addressing child care provider burnout and fostering a positive organizational climate may be important targets for future efforts aimed at reducing expulsion risk in ECE settings.

**Statement of Relevance to Infant and Early Childhood Mental Health**

This study explores factors associated with expulsion in early care and education (ECE) settings, a practice that has detrimental short‐ and long‐term effects on young children's social‐emotional development. By identifying how provider well‐being, knowledge of child development, and perceptions of organizational climate are associated with attitudes related to expulsion risk, this research seeks to identify potential areas for tailored training, mental health resources, and workplace support that may help reduce expulsion risk and promote more inclusive and developmentally appropriate early learning environments.

ECE expulsion is largely a provider‐level decision made in response to behaviors viewed as challenging (Gilliam & Reyes, 2018). That is, the decision to expel a child from an ECE setting is made by individual child care providers and/or their organization. While expulsion is a process that involves multilevel ecological systems and multiple actors, emerging studies have emphasized the importance of examining provider‐level factors associated with actual expulsion practices and providers’ decision‐making processes regarding expulsion (An & Horn, 2022; Gilliam, Maupin, Reyes, Accavitti, et al., 2016; Silver & Zinsser, 2020). Thus, further research is needed to identify the specific provider‐level factors related to expulsion decisions and practices. For this study, we focus on three key provider factors potentially associated with child expulsion from ECE settings: knowledge of child development, provider psychological well‐being, and provider perspectives of ECE organizational climate.

### 1.1 Knowledge of child development

Research has consistently shown that greater parental knowledge of child development is linked to positive child outcomes, including improved cognitive and motor skills (Dichtelmiller et al., 1992). Additionally, knowledge of child development may interact with other factors, such as maternal confidence and parental self‐efficacy, shaping how parents interpret and respond to their child's behavior and may further shape future parent‐child interactions (Conrad et al., 1992; Hess et al., 2004). The importance of knowledge of child development extends beyond parents and applies to the child care field. Studies suggest that child care providers’ understanding of child development plays a critical role in shaping their perspectives on parent‐provider relationships and communication, particularly in infant and toddler classrooms (Swartz & Easterbrooks, 2014). Bordin et al. (2000) found that while structural indicators like training and licensure were significant predictors of the quality of family child care (FCC), providers’ knowledge of child development further improved the ability to predict overall quality in FCC settings.

In addition to research supporting the importance of knowledge of child development for those in the ECE field, federal organizations in the United States have also identified a lack of early childhood educators’ knowledge of child development as a major dimension contributing to expulsion (e.g., Child Care State Capacity Building Center, 2017). Without adequate training in typical child development, teachers may misinterpret developmentally appropriate behaviors, such as toddler biting, as problematic and difficult to manage. To this point, Conners Edge et al. (2021) examined two indicators of provider knowledge: training in child social‐emotional development and program quality. They found that expulsions were more common in lower‐quality programs and in settings where teachers had not participated in state‐provided social‐emotional training. However, limited empirical research exists that more directly explores the relationship between child care providers’ knowledge of child development and their use of expulsion, particularly using established measures of child development knowledge.

### 1.2 Provider psychological well‐being

In addition to knowledge of child development, child care providers’ psychological well‐being is an important factor to consider when examining their use of expulsion in ECE settings. Previous studies have found that providers’ poor psychological well‐being, characterized by depression, stress, and emotional exhaustion, is associated with more negative interactions with children (Buettner et al., 2016). Furthermore, it is likely that the psychological load experienced by providers contributes to adverse consequences for children, as providers who are struggling with their own emotional well‐being may be more prone to perceiving children's behaviors as problematic. This has been a rich area of research; for example, teachers’ symptoms of depression have been found to be positively related to child expulsion (Silver & Zinsser, 2020). Multiple research studies have also demonstrated that teacher stress is significantly associated with preschool expulsions (Gilliam & Shahar, 2006; Loomis & Panlilio, 2022; Zinsser et al., 2019). In addition to depression and stress, provider burnout has also been found to be related to expulsion of children from ECE settings. For example, recent research by Magro et al. (2025) examined one dimension of burnout, emotional exhaustion, and found that providers who report higher levels of emotional exhaustion were more likely to expel or exclude a greater number of children from licensed child care settings.

### 1.3 Organizational climate

Importantly, factors operating at multiple levels of the social ecology have been linked to expulsion in ECE settings. In addition to provider‐level factors including knowledge of child development and psychological functioning, attributes of the child care organizations and perceptions of these attributes may also contribute to use of expulsion in ECE settings. That said, research on the impact of organization‐level factors on expulsion in ECE settings has not yielded consistent findings, likely due in part to the wide range of organizational characteristics and social relationships that form employees’ perceptions about the work environment (Peña–Suárez et al., 2013). In ECE settings, the organizational climate likely affects providers’ daily caring or teaching practices, which in turn influence classroom quality and child outcomes (Dennis & O'Connor, 2013). Miller et al. (2017) narrowed the concept of organizational climate to focus specifically on teachers’ perceptions of the availability and utility of behavioral support resources. They found that teachers’ perceptions of available resources for behavioral support were a significant predictor of preschool expulsion. Zulauf and Zinsser (2019) examined another aspect of organizational climate, finding that providers who felt supported by their preschool centers in engaging with parents were less likely to request expulsions. However, in a sample of urban preschools in Chicago, center climate did not significantly predict providers’ expulsion requests (Silver & Zinsser, 2020). Given the varying definitions of organizational climate, such mixed findings are not unexpected and suggest a need for further research to examine the relationship between the organizational climate of ECE settings and attitudes toward or use of expulsion.

### 1.4 The current study

Despite growing concerns regarding the high prevalence rate and detrimental long‐term impact of exclusionary discipline practices, limited empirical research has investigated how provider knowledge and well‐being and the child care environmental context are associated with providers’ attitudes and perceptions related to expulsion risk. Understanding these associations is important for informing policies and training programs aimed at supporting providers in managing challenging behaviors and reducing reliance on exclusionary discipline practices in ECE settings. Thus, building on existing research, the present study was designed to further examine the extent to which provider burnout, knowledge of child development, and perceptions of organizational climate, considered simultaneously, are associated with providers’ attitudes and perceptions related to expulsion risk in ECE settings.

## 2 METHODS

### 2.1 Participants

The data for the present study were drawn from the baseline phase of a longitudinal program evaluation of one child care training and technical assistance (TA) program operating in one state in the Southeastern United States. For the current study, the sample consisted of 309 child care providers in child care centers only (i.e., not including family child care homes); centers were located across the state (geographically dispersed). The majority of providers in this sample who provided information on sex (71%) identified as female. The majority of participants self‐identified as either White (35%) or African American (34%), with 66% falling within the age range of 20–60 years. Regarding professional qualifications, 52% of participants were classified as in the entry education tier in the state's child care training and workforce system (conditionally qualified or career levels 1–3), reflecting lower levels of formal ECE training and credentials. For full demographic data, see Table 1. Of note, the demographic data were only available for the group of providers as a whole, and were not collected as part of the baseline survey used in this study.

**TABLE 1:** Sample characteristics and primary variables of interest (*N* = 309).

Variable	*N* (%)	*M* (SD)	Range
Gender			
Female	218 (70.55)		
Male	2 (.65)		
Missing	89 (28.80)		
Age			
Less than 20	1 (.32)		
Between 20 and 30	77 (24.92)		
Between 30 and 40	54 (17.48)		
Between 40 and 50	35 (11.33)		
Between 50 and 60	38 (12.30)		
Between 60 and 70	30 (9.71)		
Between 70 and 80	12 (3.88)		
Missing	62 (20.06)		
Career level			
Conditionally qualified	1 (.32)		
High school diploma	74 (23.95)		
State ECE credential Level 1 (3 ECE credit hours)	67 (21.68)		
State ECE credential Level 2 (12 ECE credit hours)	9 (2.91)		
State ECE credential Level 3 (21 ECE credit hours)	11 (3.56)		
Associate's degree in a related field	1 (.32)		
Associate's degree in ECE	17 (5.50)		
Bachelor's degree in a related field	7 (2.27)		
Bachelor's degree in ECE	7 (2.27)		
Missing	115 (37.22)		
Race/Ethnicity			
Hispanic or Latino	5 (1.62)		
American Indian or Alaska Native	2 (.65)		
Asian	1 (.32)		
Black or African American	105 (33.98)		
Native Hawaiian or Other Pacific Islander	1 (.32)		
White/Caucasian	107 (34.63)		
Other	6 (1.94)		
Expulsion risk (total)		2.65 (.99)	1–5
Knowledge of child development		.82 (.21)	0–1
Provider burnout		2.48 (1.17)	1–6.4
Organizational climate		3.81 (.62)	1.4–5

*Note*: Expulsion risk (total) was assessed with the Preschool Expulsion Risk Measure (PERM). Knowledge of child development was assessed with the Knowledge of Infant Development Inventory (KIDI). Provider burnout was assessed with the Shirom–Melamed Burnout Measure (SMBM). Organizational climate was assessed with the Organizational Climate Scale (CLIOR).

### 2.2 Procedures

Child care centers were referred to receive support from the training/TA program and voluntarily agreed to participate in a one‐year program consisting of 12 group training sessions (held approximately monthly) and additional TA support between training sessions for both center directors and teachers. The research team was engaged by the training/TA program to conduct an external evaluation of program impacts over a one‐year period, with assessments designed to occur at the beginning of the program, mid‐program (6 months later), and at program close (approximately 12 months after program start). All center staff (directors and teachers) completed online assessment measures (detailed below) once the center was enrolled in the program. Data for the current study was collected at the baseline evaluation phase as centers enrolled in the training/TA program and occurred at various times during the calendar year. Measures were completed online via Qualtrics; the link for these measures was provided to the centers by their program‐assigned TA provider. This study was determined to be exempt from informed consent requirements by a University IRB.

Importantly, the state in which this study took place does not have legislation or licensing requirements banning the use of exclusionary discipline; however, the state is actively working to eliminate such practices and to improve the quality of child care services provided.

### 2.3 Measures

#### 2.3.1 Expulsion risk

The 12‐item Preschool Expulsion Risk Measure (PERM; Gilliam & Reyes, 2018) was employed to assess teachers’ attitudes and perceptions related to expulsion risk. Teachers respond to statements such as “This child's classroom behaviors interfere with my ability to teach effectively” and “My job as a teacher would be easier if this child were not in my classroom” using a 5‐point Likert scale ranging from 1 (Strongly Disagree) to 5 (Strongly Agree). The PERM includes four subscales—classroom disruption, teacher stress, fear of accountability, and hopelessness—that reflect distinct dimensions of teachers’ attitudes and perceptions related to their likelihood of using expulsion toward a child. The measure has been widely used in expulsion prevention research and evaluations in ECE settings (e.g., Gilliam, Maupin, & Reyes, 2016; Shivers et al., 2022). Gilliam and Reyes (2018) provided initial evidence for the validity of the PERM by demonstrating that higher PERM scores were associated with teachers’ consideration of permanently removing a child from their classroom and with higher teacher‐reported child behavior problems. Subsequent studies have also used the PERM as an outcome measure to capture ECE educators’ perceptions and attitudes toward young children regarding expulsion risk. For example, Zulauf‐McCurdy and Zinsser (2021) found that associations between teacher‐rated parent–teacher relationship quality and PERM scores were evident only for children without a prior expulsion history. Loomis, Musson Rose, et al. (2023) further highlighted the PERM's validity by showing that children for whom teachers reported having considered a range of exclusion practices (e.g., being kept home or termination of enrollment) had significantly higher total PERM scores; however, these findings are based on teacher‐reported consideration of expulsion rather than documented expulsion events, underscoring the need for caution when interpreting PERM as an outcome measure. The PERM has also demonstrated good internal consistency with Cronbach's *α* for each subscale ranging from .79 to .95 (Gilliam & Reyes, 2018). For the current study, providers were asked to think of a particular child in their class and to respond to the questions in the PERM measure based on that child. In this sample, the PERM demonstrated strong internal consistency (*α* = .94) and across its subscales: classroom disruption (*α* = .92), teacher stress (*α* = .89), fear of accountability (*α* = .82), and hopelessness (*α* = .84).

#### 2.3.2 Knowledge of child development

Provider knowledge of child development was assessed by asking nine questions based on the short form of the Knowledge of Infant Development Inventory (KIDI) (MacPhee, 1981). The standard KIDI short form includes 20 items (e.g., Sullivan et al., 2021). However, for the current program evaluation, a reduced set of nine key items was used to minimize survey length. This 9‐item scale assesses caregivers’ knowledge about typical infant developmental milestones and child‐rearing practices. Respondents were presented with statements, such as “If you punish children for doing something naughty, it's okay to give them a piece of candy to stop the crying,” “If children are shy or fussy in new situations, it means they have an emotional problem” and asked to choose whether they “agree,” “disagree,” or were “not sure.” A summary score for accuracy was computed by dividing the total number of items correct by the total nine questions to yield a percent score ranging from 0% to 100%. The KIDI has shown good internal reliability in two samples of mothers (*α* = .65–.67) (Bornstein et al., 2010; Hess et al., 2004). Importantly, given that the entire short form scale was not used, we did calculate internal consistency reliability of the 9‐item scale used and found it to be somewhat low but adequate (*α* = .66).

#### 2.3.3 Burnout

Burnout was assessed using the 14‐item version of the Shirom–Melamed Burnout Questionnaire (SMBM) (Shirom & Melamed, 2006). This inventory measures three key dimensions of burnout: emotional exhaustion (e.g., “I feel I am not capable of investing emotionally in coworkers and families”), physical fatigue (e.g., “I feel physically drained”), and cognitive weariness (e.g., “I feel I'm not focused in my thinking”). Participants responded to items using a 7‐point Likert scale ranging from 1 (never or almost never) to 7 (always or almost always). The SMBM has demonstrated robust psychometric properties across various occupational groups, including police officers, nurses, and healthcare workers, as well as across multiple languages, such as Chinese, French, and German (Michel et al., 2022). In this study, the scale showed high internal consistency (*α* = .93).

#### 2.3.4 Organizational climate

The organizational climate was assessed using the short version of the Organizational Climate Scale (CLIOR) developed by Peña‐Suárez et al. (2013). This 15‐item scale measures key dimensions of the workplace, such as cooperation, work organization, interpersonal relations, innovation, participation, and job attachment. Items like “Opportunities for training are offered” and “My work is adequately defined” were asked, and responses were recorded on a 5‐point Likert scale ranging from 1 (strongly disagree) to 5 (strongly agree). The scale has demonstrated strong internal consistency in various contexts, including health service workers in Spain (*α* = .94) and public hospital staff in North Cyprus (*α* = .88) (Berberoglu, 2018; Peña‐Suárez et al., 2013). The CLIOR had excellent reliability in the current sample (*α* = .93).

### 2.4 Data analysis

All statistical analyses were conducted using Stata 19. Prior to analysis, missing data and outliers were checked, and continuous variables were evaluated for skewness and potential distribution issues. First, descriptive statistics and correlation analyses were conducted on the main variables. To examine the associations between providers’ knowledge of child development, burnout, organizational climate, and attitudes related to expulsion risk, we conducted five multiple linear regression models. The first model used the total provider‐reported expulsion risk score as the outcome variable, and the remaining four models used the individual subscale scores (i.e., classroom disruption, fear of accountability, hopelessness, and teacher stress) as the outcome variable. In each model, provider burnout, knowledge of child development, and organizational climate were entered simultaneously as independent variables. Before performing linear regression, the assumptions of linearity, independence of errors, homoscedasticity, and normality were checked. The regression model did not include any covariates (see Section 4.1 for further discussion).

## 3 RESULTS

One participant with more than 50% missing values across four scales and one outlier was removed from the analysis, leaving a total of 307 participants. In total, .97% of CLIOR data and .64% of PERM data were missing; no missing values were recorded for the KIDI or SMBM scales. Descriptive statistics for study variables are reported in Table 1. Child care providers in this study reported a moderate level of attitudes and perceptions related to expulsion risk and a strong knowledge of child development. Provider burnout levels were relatively low; however, variability in responses suggests that while most providers experienced lower burnout, some reported higher levels of burnout. Organizational climate was perceived as positive overall, indicating that the majority of providers viewed their center environment favorably.

Correlations between key variables are presented in Table 2. All indicators of providers’ attitudes and perceptions related to expulsion risk were significantly correlated. Total scores for providers’ attitudes and perceptions related to expulsion risk were positively correlated with provider burnout and negatively correlated with organizational climate. Organizational climate was negatively correlated with each expulsion risk indicator and with provider burnout. Provider burnout was positively correlated with all expulsion risk indicators. Knowledge of child development was not significantly correlated with total expulsion risk or with any indicators, was not correlated with provider burnout, but was positively correlated with organizational climate.

**TABLE 2:** Correlations among study variables.

	1	2	3	4	5	6	7	8
1. Expulsion risk–Total	—							
2. Expulsion risk–Classroom disruption	.87^***^	—						
3. Expulsion risk–Fear of accountability	.88^***^	.75^***^	—					
4. Expulsion risk–Hopelessness	.81^***^	.55^***^	.61^***^	—				
5. Expulsion risk–Teacher stress	.88^***^	.68^***^	.68^***^	.68^***^	—			
6. Knowledge of child development	.01	.04	.00	–.05	.04	—		
7. Provider burnout	.46^***^	.41^***^	.40^***^	.36^***^	.41^***^	.00	—	
8. Organizational climate	–.24^***^	–.17^**^	–.20^***^	–.25^***^	–.23^***^	.17^**^	–.39^***^	—

*Note*: Expulsion risk (total) was assessed with the Preschool Expulsion Risk Measure (PERM); classroom disruption, fear of accountability, hopelessness, and teacher stress are PERM subscales. Knowledge of child development was assessed with the Knowledge of Infant Development Inventory (KIDI). Provider burnout was assessed with the Shirom–Melamed Burnout Measure (SMBM). Organizational climate was assessed with the Organizational Climate Scale (CLIOR).

* *p* < .05, ^**^ *p* < .01, ^***^ *p* < .001.

Five multiple linear regression models were conducted, and the results are presented in Table 3. Model 1, with knowledge of child development, burnout, and organizational climate as predictors of total provider‐reported expulsion risk, accounted for 22% of the variance (*R* ^2^ = .22, *p *< .001). Models 2 through 5, using the same predictors with each expulsion risk indicator as the outcome, accounted for 14%–18% of the variance (Classroom Disruption: *R* ^2^ = .17, *p *< .001; Fear of Accountability: *R* ^2^ = .16, *p *< .001; Hopelessness: *R* ^2^ = .14, *p *< .001; Teacher Stress: *R* ^2^ = .18, *p *< .001). Overall, provider burnout was positively associated with the total provider‐reported expulsion risk score (*p* < .001) and each of its four indicators: classroom disruption (*p* < .001), fear of accountability (*p* < .001), hopelessness (*p* < .001), and teacher stress (*p* < .001), indicating that higher burnout was associated with attitudes and perceptions related to the likelihood of use of expulsion across all decision‐making factors. Organizational climate was negatively associated with hopelessness (*p* = .034) but was not significantly associated with the total provider‐reported expulsion risk score or the other indicators. Knowledge of child development was not significantly associated with the total provider‐reported expulsion risk score or any indicator.

**TABLE 3:** Multiple linear regression analyses for expulsion risk.

	Expulsion risk–Total		Expulsion risk–Classroom disruption		Expulsion risk–Fear of accountability		Expulsion risk–Hopelessness		Expulsion risk–Teacher stress	
	*β*	*SE*	*β*	*SE*	*β*	*SE*	*β*	*SE*	*β*	*SE*
Knowledge of child development	.12	.25	.23	.32	.06	.30	–.16	.28	.33	.30
Provider burnout	.36^***^	.05	.41^***^	.06	.36^***^	.06	.28^***^	.05	.38^***^	.06
Organizational climate	–.14	.09	–.05	.11	–.11	.11	–.21^*^	.10	–.18	.11

*Note*: Expulsion risk (total) was assessed with the Preschool Expulsion Risk Measure (PERM); classroom disruption, fear of accountability, hopelessness, and teacher stress are PERM subscales. Knowledge of child development was assessed with the Knowledge of Infant Development Inventory (KIDI). Provider burnout was assessed with the Shirom–Melamed Burnout Measure (SMBM). Organizational climate was assessed with the Organizational Climate Scale (CLIOR).

* *p* < .05, ^**^ *p* < .01, ^***^ *p* < .001.

## 4 DISCUSSION

This study examined the relationships between provider burnout, knowledge of child development, organizational climate, and providers’ attitudes and perceptions related to expulsion risk in a real‐world sample of 309 child care providers. Findings from the multiple linear regression models demonstrated that provider burnout was significantly associated with the total provider‐reported expulsion risk score as well as each of its four indicators (i.e., classroom disruption, fear of accountability, hopelessness, and teacher stress). In line with prior research, providers with higher levels of burnout reported a greater likelihood of using expulsion (Magro et al., 2025). However, despite this apparent alignment with prior findings, consideration of how burnout is defined and assessed must be considered. Research by Magro et al. (2025) used a definition of burnout that focused solely on emotional exhaustion. An important consideration is that current research suggests that burnout is not a single–dimensional construct and the SMBM includes three components of burnout—emotional exhaustion, physical fatigue, and cognitive weariness (Shirom, 2003). However, it has been contended that SMBM captures only a general dimension of exhaustion and does not include other critical dimensions of burnout (i.e., depersonalization and reduced personal accomplishment) (Maslach & Leiter, 2016). This raises the question of what aspects of burnout may play a role in shaping providers’ decisions about how to respond to children's challenging behaviors, and thus whether or not to consider using expulsion. Furthermore, burnout and job‐related stress are often used interchangeably; thus, our findings also align with prior research linking job‐related stress to early childhood expulsion (Gilliam & Shahar, 2006; Loomis & Panlilio, 2022).

It is of interest that while burnout presence was significantly associated with expulsion risk, in our current sample, burnout was relatively low. This contrasts with prior research indicating that child care providers often reported high levels of burnout (Ansari et al., 2022; Jennings, 2015). Burnout is common in people‐oriented professions, and child care is a clear example. Systemic factors in ECE settings, such as low pay, lack of professional recognition, and high turnover (Gomez et al., 2015), may also contribute to provider psychological distress. Additionally, the day‐to‐day demands of the child care job itself, including managing children's behaviors and supporting children with special needs, can further intensify burnout among providers. One possible explanation for our finding of relatively low average levels of burnout could be sample composition. Our sample primarily included younger providers in the entry education tier of one state's child care training and workforce system, which may not fully reflect the broader experiences of the child care workforce. As such, the findings may underrepresent the complexity and prevalence of burnout among general child care providers.

In the current study, organizational climate was only significantly associated with the hopelessness subscale of the preschool expulsion risk measure (Gilliam & Reyes, 2018). This finding is consistent with prior research showing that hopelessness—a key feature of depression—is associated with expulsion (Silver & Zinsser, 2020). It is likely that providers who feel more hopeless about improving a child's behavior may hold stronger attitudes favoring exclusionary discipline practices like expulsion. However, organizational climate was not significantly associated with total provider‐reported expulsion risk score or other indicators (i.e., stress, fear of accountability, classroom disruption). One possible explanation is that the organizational climate measure used in the current study, the CLIOR scale, assesses broader, system‐level aspects of the work environment rather than more targeted supports and resources, such as center support for family collaboration (Zulauf & Zinsser, 2019) or the availability of behavioral supports (Miller et al., 2017), which may be more directly associated with providers’ attitudes and perceptions related to expulsion risk.

Finally, in the current sample, knowledge of child development was not significantly associated with providers’ attitudes and perceptions related to expulsion risk. This finding stands in contrast to previous work by Conners Edge et al. (2021), which identified general teacher preparedness in child development as a potential predictor of expulsion in a statewide expulsion prevention support system. One possible explanation is that knowledge alone, particularly when measured in a decontextualized or theoretical manner, may not translate into the practical, responsive skills needed to navigate challenging behaviors in real ECE classroom settings. Our study's measure of knowledge may not have captured applied knowledge or hands‐on skills with child‐specific developmental issues, which are likely more influential in shaping provider responses to behaviors. It could be that the quality of provider‐child interactions—how providers interpret and respond to children's needs and behaviors—is a more salient factor than knowledge per se. Simply knowing developmental milestones may be less impactful than the ability to build strong relationships, apply emotional regulation, and engage in effective classroom management strategies. This idea is supported by Loomis, Freed, et al.’s (2023) study, which found that the quality of the teacher‐child relationship mediated the effect of children's inhibitory control on providers’ attitudes and perceptions related to expulsion risk.

### 4.1 Limitations and future directions

This study is one of only a handful of studies to examine provider burnout, knowledge of child development, organizational climate, and providers’ attitudes and perceptions related to expulsion risk together in ECE settings. The findings underscore a significant association between burnout and provider‐reported expulsion risk, as well as between organizational climate and hopelessness, an indicator of the expulsion risk measure used. However, several important limitations must be noted, offering direction for future research. First, the measure of expulsion risk used in this study reflects providers’ attitudes and perceptions of expulsion risk toward a specific child, rather than objective data on exclusionary discipline or actual expulsion events. As such, we cannot determine the extent to which provider burnout is associated with actual use of expulsion. Future studies should consider linking self‐reported expulsion risk with administrative records of actual expulsions to better assess this relationship. Relatedly, the measure of burnout used in the current study has not been applied broadly in ECE populations. While this measure showed excellent internal consistency reliability in the current sample, the degree to which our finding of moderate levels of burnout is a function of the measure or of this particular sample cannot be clearly determined and may be an important focus of future work. Second, data were collected at the baseline phase of a larger program evaluation of a training/TA program that child care center directors had agreed to participate in. This sample was thus unique in that the organization had identified a need for additional support, and thus, results may not be generalizable to the universe of child care providers. In addition, for this study, we were unable to fully describe the demographic characteristics of study participants. Although providers were asked to use their unique state‐generated child care provider identification number in completing the online assessment measures, many providers did not do so, had not yet been issued the identifier, or reported the provider identification number of a colleague or supervisor. Thus, we were only able to attain aggregated demographic data from partner organizations to describe the sample generally. This prevented us from being able to control for provider characteristics such as level of education or years of experience in the field in our regression analyses. This is a notable limitation, as previous research has shown that covariates such as provider age, race, education, and years of experience (Hooper & Schweiker, 2020; Magro et al., 2025) are significantly associated with expulsion decisions and practices. Future research should examine how individual provider characteristics influence burnout, perceptions of organizational climate, and attitudes related to expulsion risk. A related limitation includes the lack of ability for our analyses to account for clustering of providers within unique child care centers. This is a critical factor for future studies, given that organizational climate does appear to be related to provider‐level characteristics (e.g., hopelessness). A final, yet important limitation to note is the cross‐sectional design of the current study, limiting the ability to draw causal inferences. For example, because data on provider burnout and attitudes and perceptions related to expulsion risk were collected concurrently, it remains unclear whether greater burnout is associated with more elevated attitudes related to expulsion risk or whether perceiving children with challenging behaviors as difficult to manage and considering expulsion, in turn, increases provider burnout. Longitudinal research is needed to explore the directionality of these relationships and to capture changes over time.

## 5 CONCLUSION

Child care providers are key actors in shaping children's early experiences and play a key role in decision‐making regarding expulsion. However, limited research has examined how individual provider knowledge, well‐being, and perceptions of organizational context may shape their attitudes regarding children's challenging behaviors that may influence decision‐making regarding expulsion. This study thus explored the relationship between these provider‐level factors: burnout, knowledge of child development, perceptions of organizational climate, and attitudes related to expulsion risk. Two notable findings emerged: provider burnout (while moderate overall) was significantly associated with providers’ attitudes and perceptions related to use of expulsion, and organizational climate was significantly associated with hopelessness (an indicator of provider‐reported expulsion risk). Provider burnout and perceptions of negative organizational climate may shape how child care providers perceive children's behavior and could possibly reduce their capacity to effectively manage challenging situations related to child behavior in the classroom. These findings suggest that it is critically important to support child care providers’ emotional and professional well‐being, along with provision of strategies for fostering supportive child care organizational environments, in efforts to reduce expulsion risk in ECE settings. Action at both the provider and organization levels of the social ecology together, along with macro‐level efforts (i.e., legislation) to eliminate expulsion in ECE settings are needed if we are to reduce this harmful practice. Although we fully acknowledge that the results obtained in this descriptive, exploratory study require replication with larger, more diverse samples and the inclusion of provider traits, this study offers a first step toward understanding how provider knowledge, well‐being, and professional context contribute to expulsion risk.

## CONFLICTS OF INTEREST STATEMENT

The authors declare no conflicts of interest.

## Data Availability

Data will be made available to the reader upon reasonable request.
